# Supplementary figures and images for: The bithorax complex iab-7 Polycomb response element has a novel role in the functioning of the Fab-7 chromatin boundary
Source: PLoS Genet. 2018 Aug 15;14(8):e1007442. doi: 10.1371/journal.pgen.1007442 (PMC6110506; doi:10.1371/journal.pgen.1007442)

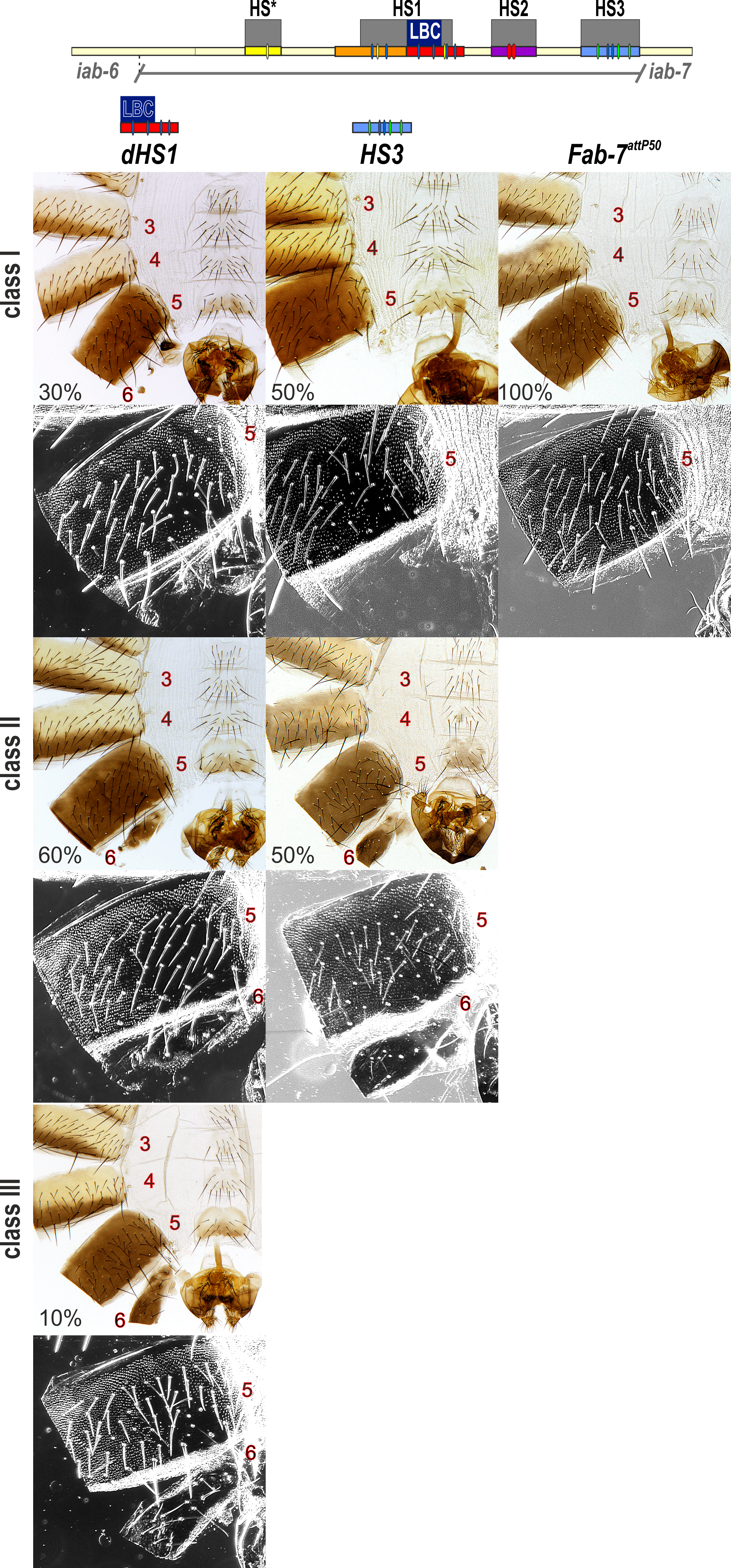

Supplement: S1 Fig — Brightfield and darkfield images of male cuticles, as indicated. dHS1: Three classes, I, II, and III, are observed. These classes differ in the size of the tergite. Class II is the most frequent. HS3: Two different classes of phenotypes are observed. The first class (I) resembles the GOF transformation of the starting Fab-7attP50 replacement platform. The second class (II) has a small residual tergite that (based on trichome hairs) appears to have an appropriate A6 (PS11) identity. Sternites are not observed in either class. Fab-7attP50: A6 is absent, indicating that PS11 is transformed into a duplicate copy of PS12. (TIF) [file pgen.1007442.s001.tif]

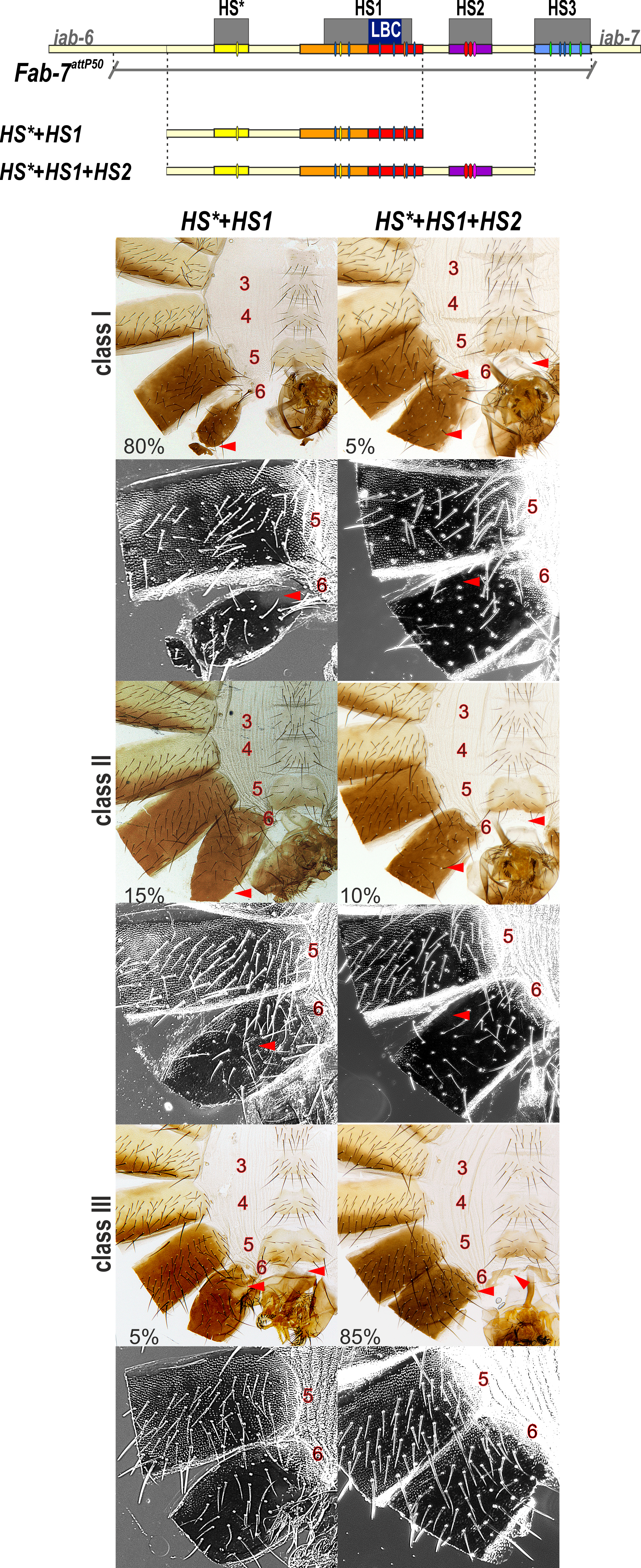

Supplement: S2 Fig — Brightfield and darkfield images of male cuticles, as indicated. HS*+HS1: The cuticular phenotypes fall into three different classes depending on the size of the A6 tergite. In the most frequent class, class I, the tergite is significantly reduced in size and misshapen. There is a modest reduction in the size of the tergite in class II, while in class III, which is the least frequent, there is only a slight reduction in the size of the tergite compared to wild type. In these flies, trichome pattern in the A6 tergite resembles that in wild type, suggesting that surviving histoblasts that give rise to the dorsal cuticle are properly specified. In all HS*+HS1 male flies the A6 sternite is missing. HS*+HS1+HS2: Three classes of cuticular phenotypes are observed. In the most frequent class, class III, the size of tergite is close to that in wild type, though sometimes the edges of the tergite are irregular. The sternite is present, but typically misshapen. Flies in the next most frequent class, class II, lack a sternite, while their tergite resembles that of class I. Finally, in class III, the tergite is noticeably reduced in size, while the sternite is misshapen. (TIF) [file pgen.1007442.s002.tif]

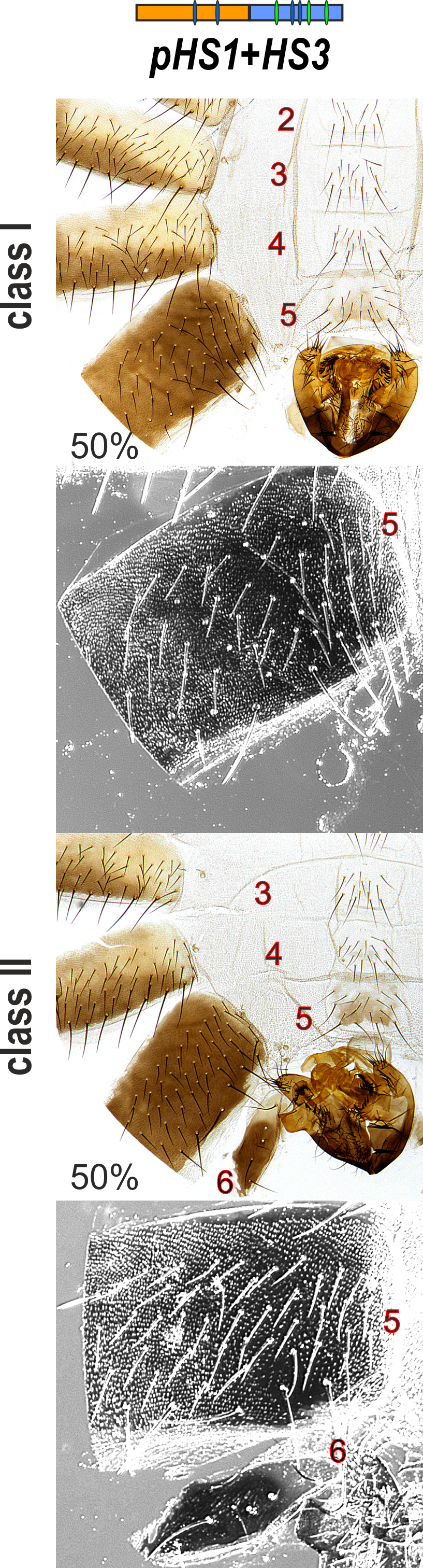

Supplement: S3 Fig — Two roughly equal classes of phenotypes are observed for the pHS1+HS3 replacement. In class I, A6 is transformed into a duplicate copy of A7, and is absent. In class II, the transformation is not complete, and a small residual A6 tergite is observed. (TIF) [file pgen.1007442.s003.tif]

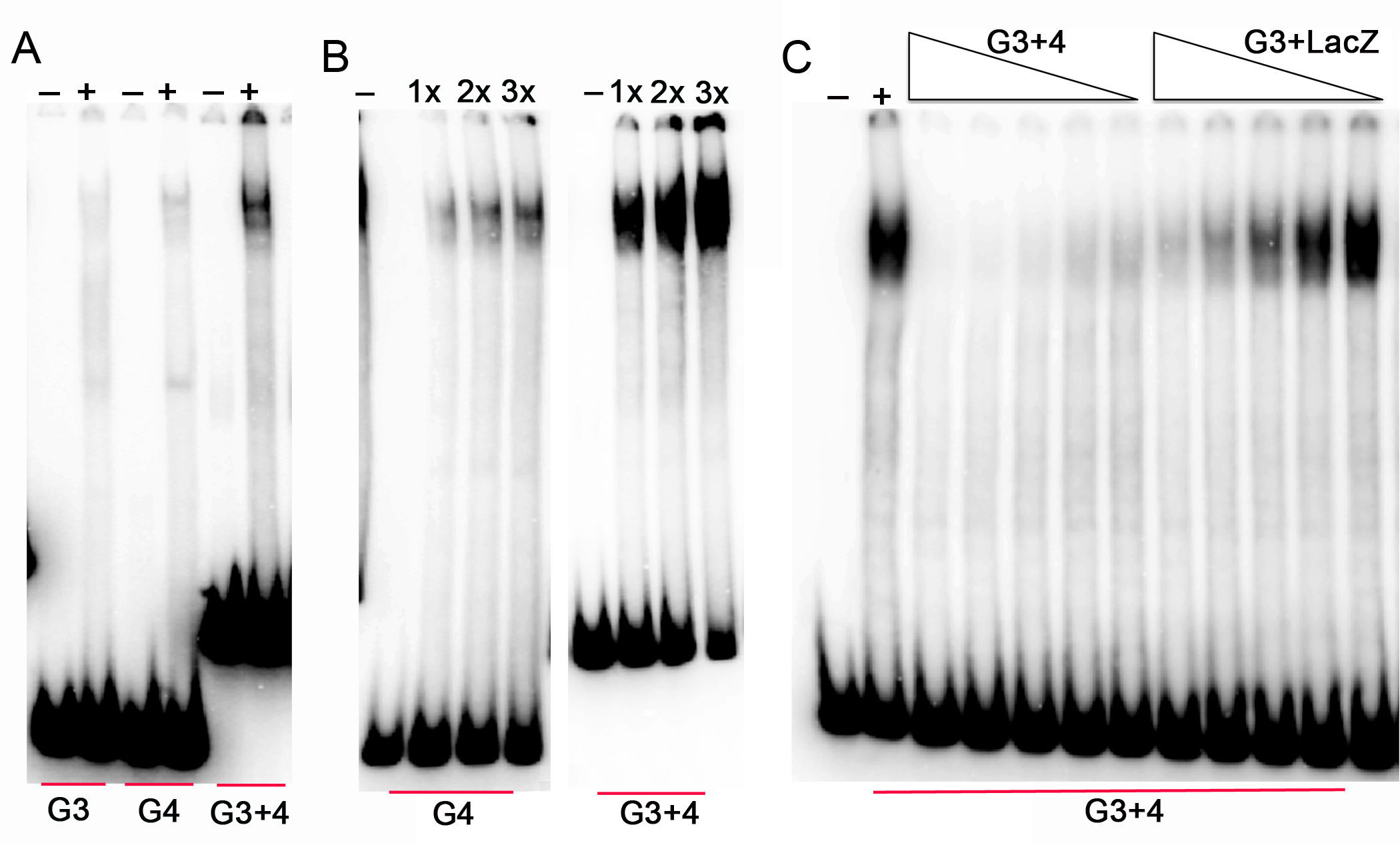

Supplement: S4 Fig — Nuclear extracts prepared from 6–18 hr embryos were used for EMSA experiments: (-) no extract, (+) with extract. Comparison of LBC binding to probes spanning just GAGA3 (G3) or GAGA4 (G4) to probes spanning both GAGA3 and GAGA4 (G3+4). (A) EMSAs of G3, G4, and G3+4. (B) EMSAs of G3 and G3+4 with increasing amount of extract (1 μl, 2 μl, 3 μl). (C) Competition experiments with probe G3+G4 and excess cold G3+G4 or G3+LacZ (left to right: 100x, 75x, 50x, 25x, and10x). (TIF) [file pgen.1007442.s004.tif]

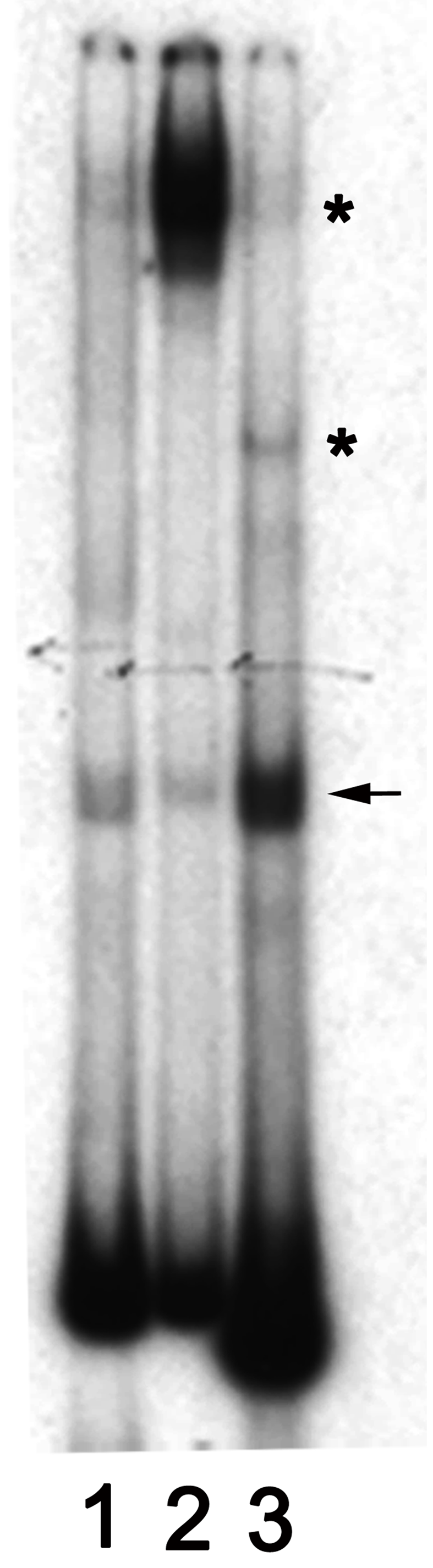

Supplement: S5 Fig — Nuclear extracts prepared from 6–18 hr embryos were used for EMSA experiments with three overlapping HS3 probes: Probe #1, 100 bp from proximal side of HS3. Probe #2, 100 bp probe from center of HS3. Probe #3, 88 bp probe from distal side of HS3. *–unique shifts; arrow–shifts observed with two or more probes. (TIF) [file pgen.1007442.s005.tif]
