# Supplementary material for: The bithorax complex iab-7 Polycomb response element has a novel role in the functioning of the Fab-7 chromatin boundary
Source: PLoS Genet. 2018 Aug 15;14(8):e1007442. doi: 10.1371/journal.pgen.1007442 (PMC6110506; doi:10.1371/journal.pgen.1007442)
Supplement: S1 Table — (PDF) [file pgen.1007442.s006.pdf]

Table S1

Primer	Sequence (5'---->3')
CHIP analysis	
1_d	GCCTCTGCTTCTGGACCTATGCTTTATT
1_r	TTTAGCCCTGCGAAGATATG
2_d	GGTGCAGTAGTAAATATAAGCAAAGAG
2_r	GCGTGCGGTTCTCTTATCAC
3_d	GTCGCAAGAACTTCACAACAG
3_r	GCCATCATGGATGTGAAAGA
4_d	TTCCGCCGACGGCATTT
4_r	GAACATCAGTTTCGTATGCGAGTTATAG
γTub37C_d	GCTTTCCCAAGAAGCTCATACA
γTub37C_r	GGTTCAGTGCGGTATTATCCAG
hsp70_d	GACAGAGTGAGAGAGCATTAG
hsp70_r	GGTTATTGTGGTAGGTCATTTG
PREeng_d	AGATGGCATGTGGCTCTCCC
PREeng_r	AACTGTGTCCCCAGCGAACTG
Probes for EMSA	
dHS1	CAAAGAGCGACACGTGAACAGGTGCAGTAGTAAATATAAGCAAAGAGAGTTGGAAAGAG<br>TATTGGCTAAGAGCGTCCGCTCACTAACACATAGATAAATTAAGAGAGACGTGATAAGAG<br>AACCGCACGCACACCACCGCAAAATCCAATTGGAAGAGAGCGACTGCTTGAGTGTATTGGT<br>TAGCAAGAGAGCGGCTAAGGTTTGATGGTTTGATTGGAATTCAGTTGCCGTTCG
dHS1 mut GAGA 3,4	ACCCTCTATCACCGTGAACAGGTGCAGTAGTAAATATAAGCAAAACAAATTGGAAAGAGT<br>ATTGGCTAAGAGCGACCGCTCACTAACACATAGATAAATTAAACAAAACGTGATAAGAG<br>AACCGCACGCACACCACCGCAAAATCCAATTGGAAGAGAGCGACTGCTTGAATGTATTGGT<br>AAGCAAGAGAGCGGCTAGGTTTGATGGTTTGATTGGAATTCAGTTGCCGTTCG
dHS1 mut GAGA3-6	ACCCTCTATCACCGTGAACAGGTGCAGTAGTAAATATAAGCAAAACAAATTGGAAAGAGT<br>ATTGGCTAAGAGCGACCGCTCACTAACACATAGATAAATTAAACAAAACGTGATAAGAG<br>AACCGCACGCACACCACCGCAAAATCCAATTGGAAACAAACGACTGCTTGAATGTATTGGT<br>AAGCAAACAAACGGCTAGGTTTGATGGTTTGATTGGAATTCAGTTGCCGTTCG
HS3	CAGACGACGTCGCAGGTGAGTGGCGAGCAGAGCAGCATGGAGCGAGCATGGCCGCTGTGGAA<br>TACCGCACTGTCGTAGGCACGAGCGCGAGCGAGAGAGGCCAAGAGCACGCTCTCTTTCACAT<br>CCATGATGGCTGCCGCTGTCTCGCCTCTTCTTCTTCATTTTCAGCTCGGCCATCATGGGGCTCC<br>ATTAAATCCACT
HS3 mut GAGA	CAGACGACGTCGCAGGTGAGTGGCGAGCAGAGCAGCATGGAGCGAGCATGGCCGCTGTGGAA<br>TACCGCACTGTCGTAGGCACGAGCGCGAGCTTTTTAGGCCAAGAGCACGTTTTTTTTCACATC<br>CATGATGGCTGCCGCTGTCTCGCCTCTTCTTCTTCATTTTCAGCTCGGCCATCATGGGGCTCCA<br>TTAAATCCACT
HS3 mut Pho	CAGACGACGTCGCAGGTGAGTGGCGAGCAGAGCAGCATGGAGCGAGCCGTACCGCTGTGGAA<br>TACCGCACTGTCGTAGGCACGAGCGCGAGCGAGAGAGGCCAAGAGCACGCTCTCTTTCACAT<br>CCATGCGTACTGCCGCTGTCTCGCCTCTTCTTCTTCATTTTCAGCTCGGTACGCATGGGGCTCC<br>ATTAAATCCACT
HS3 overlapping #1	ACGTCGCAAGAACTTCACAACAGACGACGTCGCAGGTGAGTGGCGAGCAGAGCAGCATGGA<br>GCGAGCATGGCCGCTGTGGAATACCGCACTGTCGTAGGC
HS3 overlapping #2	GTGGAATACCGCACTGTCGTAGGCACGAGCGCGAGCGAGAGAGGCCAAGAGCACGCTCTCTT<br>TCACATCCATGATGGCTGCCGCTGTCTCGCCTCTTCTT
HS3 overlapping #3	CACATCCATGATGGCTGCCGCTGTCTCGCCTCTTCTTCTTCATTTTCAGCTCGGCCATCATGGG<br>GCTCCATTAAATCCACTGCCTCTT
GAGA 3+4	CAAAGAGCGACACGTGAACAGGTGCAGTAGTAAATATAAGCAAAGAGAGTTGGAAAGAG<br>TATTGGCTAAGAGCGTCCGCTCACTAACACATAGATAAATTAAGAGAGACGTGATAAGAG<br>AACCGCACGCACACC
roX2	ACAATTGCCAAATAATACAGATCGATTTAGAGCGAGATGACAATAGAGAGGCGATCTCTC<br>TCGTATACGAGTCTTGAAAAGAAAGAGAAGGCGAACGGTGCTGGCTTAGAGAGAGATGGCA<br>ATACTAATTAACTGCAAATACATTTCCGCCATTTTGTTGGCGCTAAAAGTAACGGAAATTC<br>GAGATGCTTTTAGGGCTG
GAGA 3	CAAAGAGCGACACGTGAACAGGTGCAGTAGTAAATATAAGCAAAGAGAGTTGGAAAGAGT<br>ATTGG
GAGA 4	ATTGGCTAAGAGCGTCCGCTCACTAACACATAGATAAATTAAGAGAGACGTGATAAGAGA<br>ACCGC
GAGA 3 + lacZ	CAAAGAGCGACACGTGAACAGGTGCAGTAGTAAATATAAGCAAAGAGAGTTGGAAAGAGT<br>ATTGGGGCACCCCAGGCTTTACACTTTATGCTTCCGGCTCGTATGTTGTGTGGAATTGTGAGCG<br>GATAACAATTTCACA
